# Angulating-Distraction Ulnar Osteotomy and Interpositional Phosphocalcic Ceramic Wedge Graft for a Chronic Monteggia Lesion

**DOI:** 10.2174/1874325001711010263

**Published:** 2017-03-31

**Authors:** Jagadish Prabhu, Mohammed K. Faqi, Fahad AL Khalifa, Rashad K. Awad

**Affiliations:** Department of Orthopedics, Bahrain Defence Force Hospital – Royal Medical Services, Kingdom of Bahrain.

**Keywords:** Chronic radial head, Dislocation, Interposition graft, Monteggia lesion, Ulnar osteotomy, Wedge

## Abstract

**Background:**

Various types of osteotomies have been used to facilitate reduction of the radial head and to prevent recurrent subluxation. The Bouyala technique – open reduction of radial head associated with open wedge ulnar osteotomy with or without annular ligament reconstruction, is presently the most widely used treatment for long- standing traumatic dislocation of the radial head, independently of age, in the absence of osteoarthritis remodeling, and should preferably be performed within 1 year of trauma.

**Method:**

In this article, we present a similar case operated by same technique, but we used synthetic phosphocalcic ceramic wedge graft instead of auto bone graft as described in many other studies. We believe that, this will limit the donor site morbidity and also aid in achieving better stability at osteotomy site, which in turn help in proceeding with early active mobilization protocol.

**Result:**

We achieved union of the osteotomy by three months. Clinically, there was no deformity and she achieved full pain-free range of motion of elbow joint.

**Conclusion:**

We believe that, use of synthetic phosphocalcic ceramic wedge graft allow rigid fixation of osteotomy, provides additional stability, decrease the risk of secondary displacement and allow early mobilization, which may minimize contracture and we could achieve fairly good clinical outcome.

## INTRODUCTION

Open reduction of radial head associated with open wedge ulnar osteotomy with or without annular ligament reconstruction is presently the most widely used treatment for long- standing traumatic dislocation of the radial head, independently of age, in the absence of osteoarthritis remodeling, and should preferably be performed within 1 year of trauma [1]. An osteotomy of the ulna with over-correction and elongation aims to maintain the reduced position of the radial head through the stabilising action of the interosseous membrane. The technique requires removal of the scar tissue interposed in the radiohumeral joint, and its success depends on the absence of deformity of that joint and the avoidance of excessive pressure on the radial head after the operation. We present a case of neglected post traumatic monteggia lesion in a child treated with angulating-distaction ulnar osteotomy with allograft bone wedge interposition and open reduction of the dislocated radial head without annular ligament reconstruction.

## CASE REPORT

**Fig. (1):**
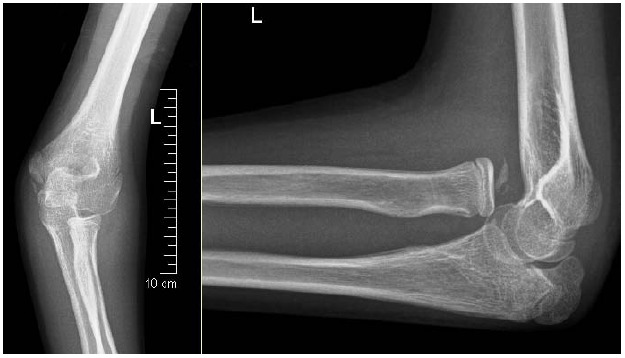
Radiographic picture of a twelve year old girl who sustained injury to her left elbow, showing anterior dislocation of the radial head (Bado type-1 lesion) with calcification of annular ligament remnants.

A 12 years old female child, sustained left elbow injury while playing. Residual displacement of the radial head had been overlooked and diagnosed as a undisplaced proximal ulna fracture in a local hospital and was immobilized in a splint for 4 weeks. Later, splint was removed and started with physiotherapy. Since child was complaining of pain with limitation of forearm rotation and difficulty in performing her routine activities, her parents approached our hospital after 8 weeks post injury. On initial examination, we noticed protrusion deformity of the radial head and cubitus valgus of 20°. The range of movement was from 25° to 110° of elbow flexion and she had mild restriction of pronation. Radiographs showed ulnar deformity and anterior dislocation of the radial head (Fig. **1**).

A surgical correction was proposed and done at 8 weeks. A pneumatic tourniquet is used with arm positioned on arn table. Gordon-Boyd approach was used to expose both the radiocapitellar joint and the proximal third of the ulna with the same incision. Fibrous tissue around the radiohumeral and proximal radio-ulnar joints is excised to facilitate the repositioning of the radial head by direct digital pressure, though at this stage the position is difficult to maintain.

A subperiostal transverse osteotomy of the ulna was performed 5 cm below the olecranon as described by Hirayama *et al.* [2]. Radial head reduction was secured and controlled by posterior angulation and distraction at the osteotomy site with aim to overcorrect the ulnar deformity. The degree of angulation and distraction was determined by evaluation of the stability of reduction of the radial head in all combinations of flexion, extension, pronation and supination under direct vision and fluoroscopy. The osteotomy was fixed with a pre-bent one-third tubular plate and 12 mm size phosphocalcic porous ceramic wedge graft (DUOWEDGE, Kasios ^®^, L’Union, France) which is composed of 60% hydroxyapatite and 40%. We trimmed this wedge with motorized saw blade, according to measured osteotomy gap size. It is important to ensure that the repositioned radial head lies in the radial notch of the ulna; this ensures proper spacing in the radiohumeral joint and prevents excessive pressure on the radial head. Postoperatively, the elbow is immobilised in 90° flexion and full supination in a plaster splint. Active movements started after two weeks.

**Fig. (2):**
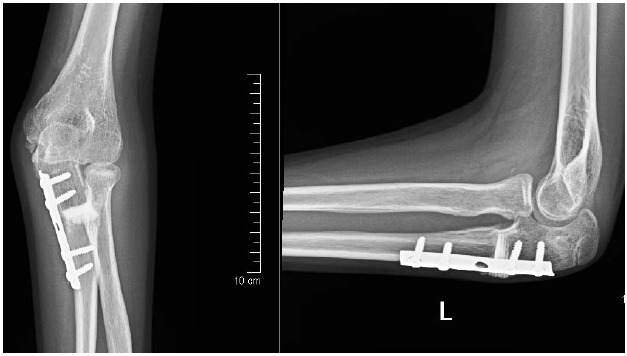
Radiograph one year after open reduction of the radial head with angulating-distraction ulnar osteotomy, showing well reduced radial head and incorporation of interpositional phosphocalcic ceramic wegde graft.

**Fig. (3):**
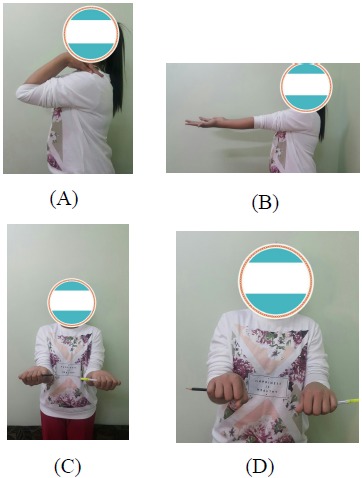
Clinical and functional results of the patient at one year follow-up.

We achieved union of the osteotomy by three months. At one year follow-up, there was some posterior convexity persisted at the site of the ulnar osteotomy but the radial head was normally located and the distal radio-ulnar joint appeared to be normal (Fig. **2**). Clinically, there was no deformity and she achieved full pain-free range of motion of elbow joint (Fig. **3**).

## DISCUSSION

Various types of osteotomies have been used to facilitate reduction of the radial head and to prevent recurrent subluxation. They include floating osteotomy without fixation or stabilized by graft, corrective diaphyseal osteotomy, proximal bending osteotomy, angulation and elongation osteotomy, gradual lengthening and angulation of the ulna using an external fixator [3-12].

Ulnar lengthening permits reduction, providing sufficient place for the radial head while avoiding excessive pressure on the radiocapitellar joint and the angulation creates an overcorrection and tensioning of the interosseous membrane which firmly maintains the head in place for the time necessary for its stabilization [3, 7]. The osteotomy of the proximal ulna with both angulation and elongation allows stable radial head reduction without necessity of annular ligament reconstruction in most of the cases.

We used, synthetic 12 mm size phosphocalcic ceramic wedge graft (DUOWEDGE, Kasios ^®^, L’Union, France), which is mainly used for high tibial osteotomy. But we found that this wedge graft is very useful even for such cases because of the following features: it has dual porosity, compressive strength greater than 80 MPa, totally interconnected porosity, sterile, ready for use, 100% synthetic, no risk of cross-contamination, calcium phosphate, and tri calcium phosphate (TCP) enhances osseointegration process [13].

In this study, we used biphasic porous ceramic material which is mainly composed of hydroxyl-apatite and tricalcium phosphate. Ceramics offer desirable characteristics such as bio-compatibility for use as bone implants, chemical inertness in biological mediums and hardness, but they have low resistance to traction. This ceramic wedge, which was placed at the osteotomy bone gap, functioned as a support for bone tissue regeneration. Thus, it allowed the regeneration tissue to grow within its physical structure because of the presence of pores, thus avoiding encapsulation due to fibrous connective tissue and increasing the speed of tissue growth. Synthetic calcium phosphate bone grafts have both osseointegration and osteoconductive properties. Osseointegration results from formation of a layer of hydroxyapatite after implantation.

In our case, ulnar osteotomy was internally fixed with a pre-bent plate and screws along with interpositional allograft bone wedge instead of tricortical autobone graft which makes this procedure quick without bone graft donor site morbidity.

## CONCLUSION

Treatment of bone defect in angulation–distraction osteotomy for chronic montaggia lesion was shown in this study, through the use of phosphocalcium porous ceramic wedge graft composed of hydroxyapatite and tricalcium phosphate, to be a practical, effective and safe method. We believe that, use of synthetic phosphocalcic ceramic wedge graft allows rigid fixation of osteotomy, thus providing additional stability, decreasing the risk of secondary displacement, and allowing early mobilization, hence one can achieve fairly good clinical outcome.
